# Cognitive style in anorexia nervosa: a preliminary investigation

**DOI:** 10.1186/2050-2974-3-S1-O63

**Published:** 2015-11-23

**Authors:** Natalie Crino, Stephen Touyz

**Affiliations:** 1 Department of Medical Psychology, Westmead Hospital, Sydney, Australia; 2 The University of Sydney, Sydney, Australia

## 

Considerable research has been devoted to examining the content of cognition in eating disorders, which typically includes dysfunctional thoughts, attitudes and underlying assumptions about the importance of controlling eating, shape and weight. Far less is known about the style of cognition. An unhelpful cognitive style might include perseverative forms of thought (Nolen-Hoeksema, Parker & Larson, 1994); problem-oriented coping (Carver, Scheier & Weintraub, 1989); the tendency to become over-involved or “fused” with cognition (Hayes, Luoma, Bond, Masuda, & Lillis, 2006); and having a self-blaming attributional style (Anderson, Miller, Riger & Sedikides, 1994). The aim of the current study was to examine the extent to which aspects of cognitive style are associated with symptoms and features of anorexia nervosa. Participants from a specialist eating disorder clinic completed self-report questionnaires examining cognitive content, cognitive style, and symptoms. Preliminary findings demonstrate strong inverse relationships between eating disorder symptoms and reappraisal, problem-oriented coping, and cognitive defusion in particular. These results highlight the need to further investigate the role of cognitive style in anorexia nervosa. This aspect of cognition might help to explain why cognitive pathology tends to persist even in those who have recovered.

